# Immune cell populations in the tumour environment following calcium electroporation for cutaneous metastasis: a histopathological study

**DOI:** 10.2340/1651-226X.2024.19462

**Published:** 2024-05-28

**Authors:** Mille Vissing, Sandra Sinius Pouplier, Lars Munch Larsen, Stine Krog Frandsen, Alexey Lodin, Anne-Vibeke Lænkholm, Julie Gehl

**Affiliations:** a Centre for Experimental Drug and Gene Electrotransfer (C*EDGE), Department of Clinical Oncology and Palliative Care, Zealand University Hospital, Næstved, Denmark; b Department of Clinical Medicine, Faculty of Health and Medical Sciences, University of Copenhagen, Copenhagen, Denmark; c Department of Surgical Pathology, Zealand University Hospital, Roskilde, Denmark

**Keywords:** Calcium electroporation, cutaneous metastases, histopathology, cancer immunity, tumour microenvironment, clinical trial

## Abstract

**Background and Purpose:**

Calcium electroporation (CaEP) involves injecting calcium into tumour tissues and using electrical pulses to create membrane pores that induce cell death. This study assesses resultant immune responses and histopathological changes in patients with cutaneous metastases.

**Patients/Materials and Methods:**

The aimed cohort comprised 24 patients with metastases exceeding 5 mm. Tumours were treated once with CaEP (day 0) or twice (day 28). Biopsies were performed on days 0 and 2, with additional samples on days 7, 28, 30, 35, 60, and 90 if multiple tumours were treated. The primary endpoint was the change in tumour-infiltrating lymphocytes (TILs) two days post-treatment, with secondary endpoints evaluating local and systemic immune responses via histopathological analysis of immune markers, necrosis, and inflammation.

**Results:**

Seventeen patients, with metastases primarily from breast cancer (14 patients), but also lung cancer (1), melanoma (1), and urothelial cancer (1), completed the study. Of the 49 lesions treated, no significant changes in TIL count or PD-L1 expression were observed. However, there was substantial necrosis and a decrease in FOXP3-expression (p = 0.0025) noted, with a slight increase in CD4+ cells but no changes in CD3, CD8, or CD20 expressions. Notably, four patients showed reduced tumour invasiveness, including one case of an abscopal response.

**Interpretation:**

This exploratory study indicates that CaEP can be an effective anti-tumour therapy potentially enhancing immunity. Significant necrosis and decreased regulatory lymphocytes were observed, although TIL count remained unchanged. Several patients exhibited clinical signs of immune response following treatment.

## Introduction

Calcium electroporation (CaEP) is a minimally invasive cancer treatment. It involves injecting calcium into cancer tissue and applying short, high voltage, pulsed electric fields (100 μs, 1,000 V/cm, and 1 Hz), with a needle electrode. This creates temporary pores in treated cell membranes, facilitating uptake of toxic levels of calcium in targeted cancerous tissue. This disrupts cancer cell homeostasis and metabolism, resulting in necrosis and rapid cell death [1–3].

CaEP exhibits a distinctive characteristic of inducing necrotic cancer-cell death in therapeutic doses (around 220 mM) when combined with the above-mentioned pulse-parameters [1, 4]. Preclinical and clinical studies have identified that CaEP treatment leads to a release of damage-associated molecular patterns (DAMPs) from necrotising cancer cells, including adenosine-triphosphate (ATP) and high-mobility group box 1 (HMGB1) [1, 5–7]. A recent study has described the activation of membrane sodium channel TRPM4 by osmotic stress from calcium ions. The resulting influx of sodium ions causes the cell to swell, which combined with ATP depletion, leads to necrosis and release of DAMPS [8]. These DAMPs attract antigen presenting cells (APCs) and induce recruitment of tumour infiltrating lymphocytes (TILs) [9], and thus CaEP may potentially initiate systemic immunity *in vivo* [6]. Additionally, recent studies have shown that CaEP, with or without pro-inflammatory interleukin-12 gene electrotransfer, can exhibit effective responses against tumours, with varying outcomes depending on tumour type and immune system involvement [10]. These findings underscore the immunogenic potential of CaEP and its possible impact on the cellular dynamics of the tumour microenvironment.

Interestingly, a recent study pinpoints the importance of necrosis in immune response, and lists treatments which lead to elevated intracellular calcium and tumour necrosis, including CaEP [8].

Clinically, CaEP has been shown to be safe and effective in treating cutaneous and mucosal malignancies, with some trials reporting long-term and abscopal responses. These findings suggest a potential synergistic effect with the immune system [11–16]. Notably, Jensen et al. reported a case of long-term disease control with repeated CaEP treatments of cutaneous metastases in human epidermal growth factor receptor 2-positive (HER2-positive) breast cancer [16], and Falk H. et al. demonstrated the induction of a systemic immune response and distant tumour remission in a patient with melanoma, triggered by CaEP [12].

Numerous studies have demonstrated the prognostic value of TILs and programmed death-ligand 1 (PD-L1) expression in various cancer types [11, 17]. PD-L1 is a protein found on cancer cells that regulates immune responses by interacting with PD-1 on immune cells. Elevated levels of PD-L1 help cancer evade immune detection [18]. When TILs express PD-1, they may contribute to inhibition of anti-tumour immune responses [19]. Targeting PD-1 with immune checkpoint inhibitors show promise in improving treatment outcomes [20]. Investigating the changes in TIL infiltration and PD-L1 expression following CaEP treatment in cutaneous metastases could provide insights into histopathological mechanisms of action. This could help identify patients who may benefit from a combination of CaEP and immunotherapy agents targeting the PD-1/PD-L1 pathway [18, 19]. In breast cancer, higher levels of TILs in estrogen receptor-negative (ER-negative) and HER2-positive subtypes are associated with better prognoses [21, 22]. In contrast, the prognostic impact of TILs appears to be reversed in ER+ breast cancer [23].

The aim of this study was to elucidate the histopathological changes induced by CaEP treatment in patients with cutaneous metastases of different cancer types and to explore potential associations with immune responses. The hypothesis is that CaEP induces tumour cell necrosis, which enhances immune cell infiltration and stimulates an immunological response in treated areas.

## Materials and methods

### Study design and setting

This trial was a non-randomised phase II study, exploring the histopathological effects of CaEP in patients with cutaneous metastases. Participants were recruited and treated at the Department of Oncology, Zealand University Hospital (ZUH). The trial was approved by the Danish Medicine Agency, The Regional Ethics Committee, and the Danish Data Protection Agency. The study was performed and monitored according to Good Clinical Practice (GCP) and registered on ClinicalTrials.gov (NCT04259658). All patients provided written informed consent.

### Ethics and dissemination

The CaEP-B study is approved by the European Union Drug Regulating Authorities (EudraCT no. 2019-004315-31), the Danish Data Protection Agency (REG-114-2019) and Ethics committee for Region Zealand (SJ-811), and registered on Clinicaltrials.gov (NCT04259658). This study adhered to the Declaration of Helsinki’s ethical principles. Participants provided informed consent and participant well-being, safety, and confidentiality were prioritised. Participants provided consent to the investigators for this publication.

### Data availability

De-identified participant data can be obtained from the corresponding author upon reasonable request. Due to patient privacy requirements, the biological data generated in this study are not publicly accessible. The corresponding author can provide these data upon reasonable request, subject to approval from the relevant ethics committee. Additional data generated in this study can be found within the article and its supplementary data files.

*Participants.* Adult patients with histologically verified cutaneous or subcutaneous primary or secondary cancer of any histology, with performance status ≤2 (Eastern Cooperative Oncology Group/World Health Organization) and at least one cutaneous or subcutaneous tumour measuring a minimum of 5 mm were eligible to participate.

Participants were allowed to undergo simultaneous medical treatment (endocrine therapy, chemotherapy, immunotherapy, etc.) including radiation therapy, not involving the CaEP target area, during the study period. All patients followed standard of care and had been offered all available alternatives before entering the protocol. Patients were treated once, and those with more than four metastases were offered retreatment after 4 weeks (Supplementary Table S1). Participants were followed with regular examinations for 3 months, with optional visits after nine and 12 months.

*Calcium electroporation.* CaEP was performed under local anaesthesia (lidocaine with epinephrine) based on ESOPE guidelines [20]. The method is presented in detail in an associated protocol article [24]. The target area (tumour tissue) including a 3 mm margin was manually injected with calcium (220 mM) followed by manual electroporation (8 pulses of 100 μs, 1000 V/cm, and 1 Hz) using handheld linear array electrodes [24]. and a Cliniporator pulse generator (IGEA, Carpi, Italy). Up to eight metastases per patient were treated. The calcium chloride dose was based on tumour size with 0.5 ml/cm³ for tumours >5 mm and 1 mL/cm³ for tumours ≤5 mm [13, 15]. Tumour volume was calculated using the formula *ab*²*π*/6 (*a* = largest diameter and *b* = diameter perpendicular to *a*). Patients could be treated more than once in previously treated areas after 2 months and could request treatment of more lesions. The results from additional targets were not followed in the study but were noted in the patient journal.

*Response*. Response was defined as change in diameter of total tumour in target areas from baseline, similar to Response Evaluation Criteria in Solid Tumors (RECIST), measured with a ruler and documented by digital photography [24]. Clinical response refers to macroscopic tumour reduction.

*Sample collection.* As it has been shown that CaEP induces rapid necrotic cell death, punch biopsies (4 mm) from the target area were performed before treatment and on day two, based on preclinical studies [1]. If more than one metastasis was treated, additional biopsies were taken at different time points after treatment (day 7, 28, 30, 35, 60, and 90) (see Supplementary Table S1). Samples were placed in formalin for preservation immediately after collection, and after 24 h of fixation, they were processed for paraffin embedding and storage as formalin-fixed and paraffin embedded (FFPE) tissue blocks.

*Histopathology analysis.* Histopathological analyses were performed to evaluate immune markers and morphological changes. Haematoxylin and eosin (HE) staining was performed on the FFPE samples to evaluate morphological changes within the tumour microenvironment. Mitotic activity, nuclear swelling, inflammation, necrosis, and the presence of TILs were assessed from HE-stained sections. In HE-stained samples, highly eosinophilic areas of amorphous tissue were interpreted as tissue damage and necrosis. Estimation of TILs were performed according to the 2014 recommendations, see below [21]; Immunohistochemical (IHC) analyses were performed according to standardised procedures, including PD-L1, HER2, ER, B-cell antigen (CD20), T-cell co-receptor CD3 (T-helper and cytotoxic T-cells), T-cell co-receptor CD4 (T helper cells, monocytes, macrophages, and dendritic cells), CD8 (cytotoxic T-cells), and FOXP3 (regulatory T-cells). The following reagents (anti-body clones) were applied for IHC-staining: JC/70A (CD31); LN10 (CD3); EP204 (CD4); C8/144B (CD8); L25 (CD20); 236A/E7 (FOXP3); SP1 (ER); 4B5 (HER2). The SP142 assay was applied for PD-L1 with interpretation of staining reaction in immune cells as recommended in the Impassion130 trial [18]. The stained slides were examined under a light microscope or from scans (Hamamatsu S360MD slide scanner with NDP-viewer software). An experienced pathologist and a resident in pathology, blinded to the clinical data, evaluated the results.

*Quantification.* TILs were quantified as area of intra-tumoral stromal tissue occupied by mononuclear inflammatory cells as opposed to the total area of intra-tumoral stroma in increments of 5%. Mitosis, necrosis, inflammation, nuclear swelling, and elastoid degeneration were scored as either 1 for present or 0 for absent. The degree of carcinoma was quantified as a percentage of viable tumour cells in the sample. PD-L1 expression was quantified with a cut-off at 1% of positive immune cells [18]. ER was quantified with a cut off at 1% positive tumour cells, and HER2 was scored 0–3 according to ASCO CAP guidelines [25, 26]. CD3, CD4, CD8, CD20, and FOXP3 were evaluated as a global score [27].

### Trial outcomes

The primary endpoint was difference in TIL population in treated cancer tumours two days after CaEP compared to before treatment. TIL content in biopsies was assessed pathologically from biopsy samples. Secondary endpoints involved investigation of the adaptive tumour immune response, assessment of clinical treatment response, evaluation of the relationship between TIL population changes and tumour type, examination of regressive changes and PD-L1 expression. Adverse events were recorded using Common Terminology Criteria for Adverse Events version 4.0 (NCI-CTCAE 4.0).

### Statistics

The study assessed changes in TILs two days after treatment using paired *t*-test, with a target of 24 patients for feasibility and representation of cancer types. Treatment response, lymphocyte subpopulations, PD-L1 expression, necrosis, and inflammation were evaluated at different time-points post-treatment. Linear and logistic regression models were used to explore potential biomarkers associated with treatment response. Descriptive statistics were reported, and a *p*-value of <0.05 was considered statistically significant, with Bonferroni correction for multiple testing where relevant (change in lymphocyte subpopulations).

## Results

### Participants

A total of 17 patients (median age 65± years [range 41–88], 88% female) with disseminated stage IV cancer disease were included. The primary diagnosis was breast cancer in 14 (82%) patients, and lung cancer, melanoma, and urothelial cancer in the remaining three patients. Concomitant chemotherapy was received by 10 patients (59%), while 4 (24%) received immune- or targeted therapy. Median time since primary cancer diagnosis was 1.8 years (range 0.5–19.2). Median follow up time was 2 months (range 0–12). Five patients (29%) went off-study before 3 months follow up due to primary disease progression (Supplementary Figure S1). Demographic, clinical and histopathological are reported for study participants in Table 1.

**Table 1:** Participant details.

Pt. No.	Age Sex	Primary tumour	Pathology	Included tumour localisation(s)	Years since diagnosis	Previous treatment	Concomitant oncological treatment	No. of incl. lesions
1	69	Breast cancer	ER pos 100%	Trunk front	2.8	No surgery (auto mastectomy)	Endocrine therapy – *Fas*	3
	F		HER2 norm			Endocrine therapy *– Let, Exe*		
						Radiotherapy		
2	68	Breast cancer	ER pos 15%	Trunk front	4.8	Surgery	Endocrine therapy – *Let*	4
	F		HER2 high			Chemo *– Cap*		
			Ki-67 >20%			Radiotherapy		
						Immunotherapy – Tras, Pert, TDM1		
3	56	Breast cancer	ER pos 25 %	Trunk front	2.5	Surgery	Chemo – *Eri*	2
	F		HER2 norm	Trunk Back		Chemo *– Cap*		
			PD-L1 3%			Radiotherapy		
4	76	Breast cancer	ER neg	Trunk front	1.8	Endocrine therapy – *Tax*	None	1
	F		HER2 norm			Chemo *– Cap, Eri*		
			Ki67 90%			Radiotherapy		
5	88	Breast cancer	ER pos 90%	Trunk front	16.3	Endocrine therapy *– Let, Ana, Exe, Ful*	Endocrine therapy – *Ana*	2
	F		HER2 norm	Upper extremity				
			Ki67 30%					
6	76	Breast cancer	ER pos 60%	Trunk front	1.1	Surgery	Chemo – *Cap*	2
	F		HER2 norm			Endocrine therapy – Let, Tam		
						Chemo – EC, Pac		
						Radiotherapy		
7	68	Breast cancer	ER pos 30%	Trunk front	1.1	Surgery	Alendronic Acid	3
	F		HER2 norm			Chemo *– Doce, Vino, Cap – Eri, CMF*		
			PD-L1 1%			Endocrine therapy – Let		
						Radiotherapy		
						Immunotherapy – Herc, Per, TDM-1		
8	56	Breast cancer	ER neg	Trunk front	1.2	Surgery	Chemo – *Eri*	1
	F		HER2 norm			Chemo – Epi, Cyc, Pac, Car, Gem, Eri		
9	54	Breast cancer	ER neg	Trunk front	4.2	None	Chemo – *Cap*	1
	F		HER2 norm					
			Ki-67 70%					
10	66	Breast cancer	ER neg	Trunk front	15.6	Chemo – *Doce, Cyc, Epi, Vino*	Chemo – *Vino*	4
	F		HER2 high			Immunotherapy – *TDM-1, Tras, Per*		
						Radiation therapy		
11	73	Urothelial cancer	Transitional cell carcinoma	Trunk front	1.8	Surgery	Immunotherapy – *Pembro*	1
	M		PD-L1 neg			Chemo *– Carb, Gem*		
						Immunotherapy – Pembro		
						Radiotherapy		
12	65	Lung cancer	Adenocarcinoma	Trunk back	0.5	Chemo – *Car, Peme*	*Prednisolone*	1
	F		PD-L1 1–25%					
13	83	Breast cancer	ER neg	Trunk front	1	Surgery	Chemo – *Eri*	4
	F		HER2 high			Chemo – *Vino, Eri*	Antibody therapy – *Tras*	
						Radiotherapy		
						Immunotherapy *– Tras*		
14	47	Melanoma	Superficial	Head and neck	0.3	Surgery	Chemo – *Temo*	6
	M		Clark’s level 2			Radiotherapy	Prednisolone	
			BRAF-mut			Immunotherapy – *Nivo, Eri, Ipi, Pembro*	Midazolam	
			PD-L1 1–5%					
15	66	Breast cancer	ER neg	Trunk front	19.2	Surgery	Chemo – *Eri*	8
	F		HER2 borderline			Radiotherapy	Antibody therapy – *Tras*	
			Ki67 10%			Chemo – *Nav*		
						Immunotherapy *– Herc*		
16	41	Breast cancer	ER pos5%	Trunk front	0.8	Chemo *– Gem*	Chemo *– Gem*	4
	F		HER2 norm					
			PD-L1 1%					
17	61	Breast cancer	ER pos 100%	Trunk front	3.2	Surgery	Chemo – Eri, Immunotherapy – Deno	2
	F		HER2 norm			Chemo *– Cap, Pal, Eri*		
			Ki67 50%			Radiotherapy		
						Endocrine therapy *– Let, Ana*		

M: Male; F: Female; pos: Positive; neg: Negative; norm: Normal; ER: Estrogen receptor; HER2: Human epidermal growth factor receptor 2; Ki-67: Antigen KI-67; PD-L1: Programmed death-ligand 1; Chemo: Chemotherapy; Neoadj: Neoadjuvant; Adj: Adjuvant; Ana: Anastrozole; Cae: Caelyx; Cap: Capecitabine; Car: Carboplatin; Cis: Cisplatin; CMF: Cyclophosphamide + methotrexate + fluorouracil; Deno: Denozumab; EC: Epirubicin + Cyclophosphamide; Eri: Eribulin; Fas: Faslodex; Fol: Folfirinox; Ful: Fulvestrant; Gem: Gemcitabine; Let: Letrozole; Pac: Paclitaxel; Pal: Palbociclib; Pembro: Pembrolizumab.

### Tumour characteristics and delivered treatment

A total of 49 tumours (median two per patient [range 1–8]), with a median size of 20 mm (7–63) were treated. The largest and/or most symptomatic area was defined as the primary treatment target (target 1) (Supplementary Figure S2). Baseline samples were only taken from target one. A total of 108 biopsies were harvested from 44 targets in 17 patients (median five [IQR 3–7] biopsies per participant). For an overview of sampled targets see Supplementary Figure S7.

One patient (pt. 15) had four tumours retreated day 28 (8.2% of all samples). Tumour and treatment characteristics are presented in Table 2.

**Table 2:** Tumour characteristics and treatment.

Tumour characteristics and treatment			
	*n*	range	%
Total treated tumours, *n*	49		
Median no. of treated lesions per patient, *n* (range)	2	1–8	
Tumour size (baseline)			
Median volume, cm^3^	4.2	0.2–66.8	
Median largest diameter, mm (range)	20	7–63	
Tumour type			
Breast cancer	41		83.7
Melanoma	6		12.2
Lung cancer	1		2.0
Urothelial cancer	1		2.0
Anatomic location			
Trunk	41		83.7
Head and neck	6		12.2
Upper extremity	1		2.0
Lower extremity	1		2.0
Previous radiation			
Non-irradiated, *n* (%)	20		40.8
Irradiated, *n* (%)	29		59.2
Calcium electroporation treatment			
Median injected dose of calcium chloride 220 mM, mL (range)*	3.9	0.2–40	
Median current (A)*	9.5	3.5–21.5	

* First treatment (see Results – Tumour characteristics and delivered treatment).

### Change in tumour infiltrating lymphocytes (primary outcome) and subpopulations

**Figure 1:**
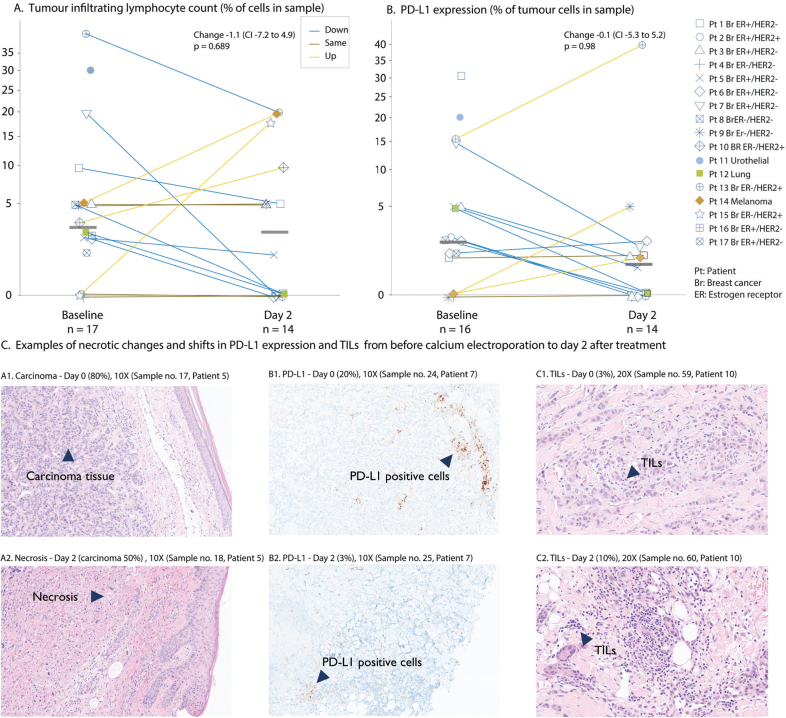
TIL count and PD-L1 expression day 0–2 from cutaneous metastases treated with calcium electroporation, at patient level. (A) TILs quantified as percentage of tumour infiltrated by lymphocytes shown on a square root scale. Median change –1.1 (95% CI –7.2 to 4.9), p = 0.69. (B) PD-L1 expression quantified as percent positive immune cells in sample. Change –0.1 (CI -5.3 to 5.2), p = 0.98. Samples taken from day 0 are baseline control samples taken before treatment. Samples were taken from tumour lesions. Different colour symbols represent tumour type, and each dot is a sample. The crossbar represents the median count for each time point. Symbols with colours according to tumour types: breast cancer (dark blue symbols); urothelial cancer (light blue); melanoma (orange); lung cancer (green). Pt. 11 and 17 day two samples not available for analyses. (C) Day 0–2: Histological changes from baseline. Examples of induced necrosis (HE-staining, A1–2), change in PD-L1 (SP142 Ventana staining, B1–2) and TILs (HE-staining, C1–2).

No significant change in TIL count was observed two days after CaEP (Mean change –1.1%, [CI –7.2 to 4.2], *p* = 0.689) (Figure 1). No correlation was observed between TIL count and proportion of viable tumour cells. Pre-specified secondary analysis of the status and change in lymphocyte subpopulations over time are graphically represented in Figure 2. CD4-positive lymphocytes were the dominant lymphocyte subpopulation throughout the study (Figure 2A). When evaluating the change in expression of lymphocyte surface markers over time, most remained static during the study, although the number of evaluated samples were small after day 2 (Figure 2B). However, a significant decline in expression of FOXP3 was observed on day 2 (Bonferroni corrected *p* = 0.0125), and a trend towards an increase in CD4 was observed on day 7 after treatment (*p* = 0.10).

**Figure 2A:**
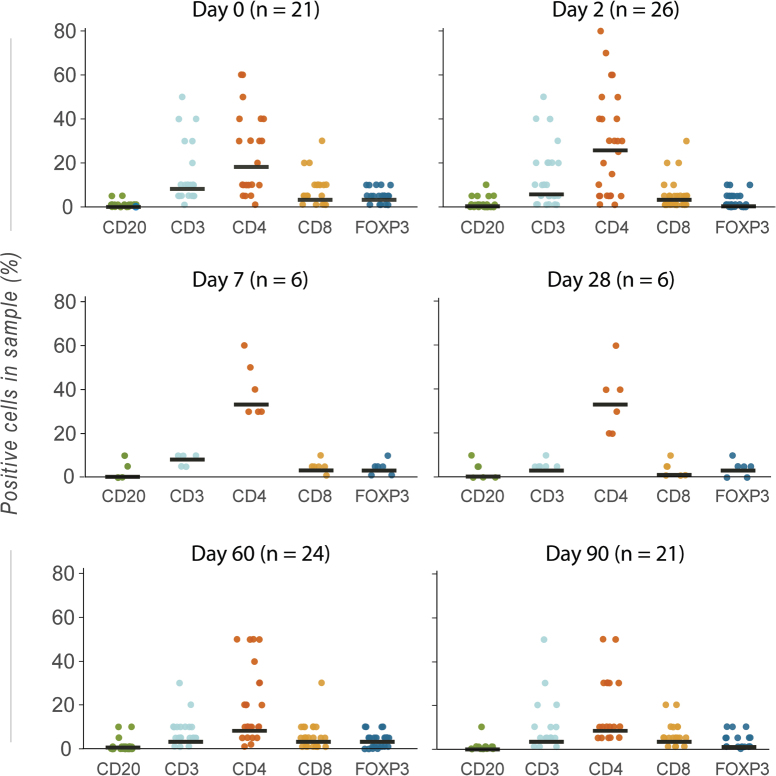
Change in lymphocyte subpopulations in samples from cutaneous metastases treated with calcium electroporation day 2–90, from baseline. (A) Lymphocyte immune markers across all samples. CD3 (total T cells); CD4 (T-helper cells, monocytes, macrophages, and dendritic cells); CD8 (effector T cells); CD20 (B cells); FOXP3 (Tregs). The median is marked with a black line. Values are percentage of total sample.

**Figure 2B:**
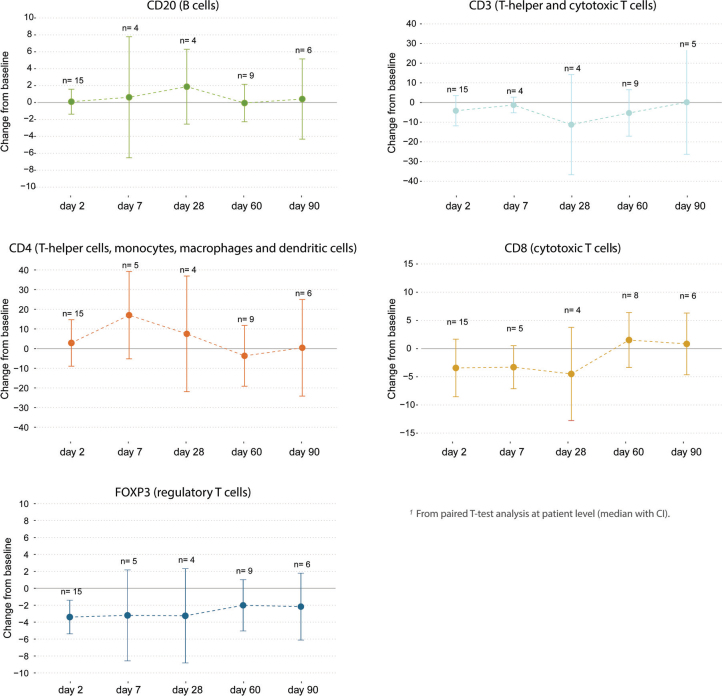
Change from baseline at patient level for analysed markers with associated lymphocyte population. From paired t-test analysis (median estimates with CI).

### Necrosis and inflammation following CaEP

**Figure 3:**
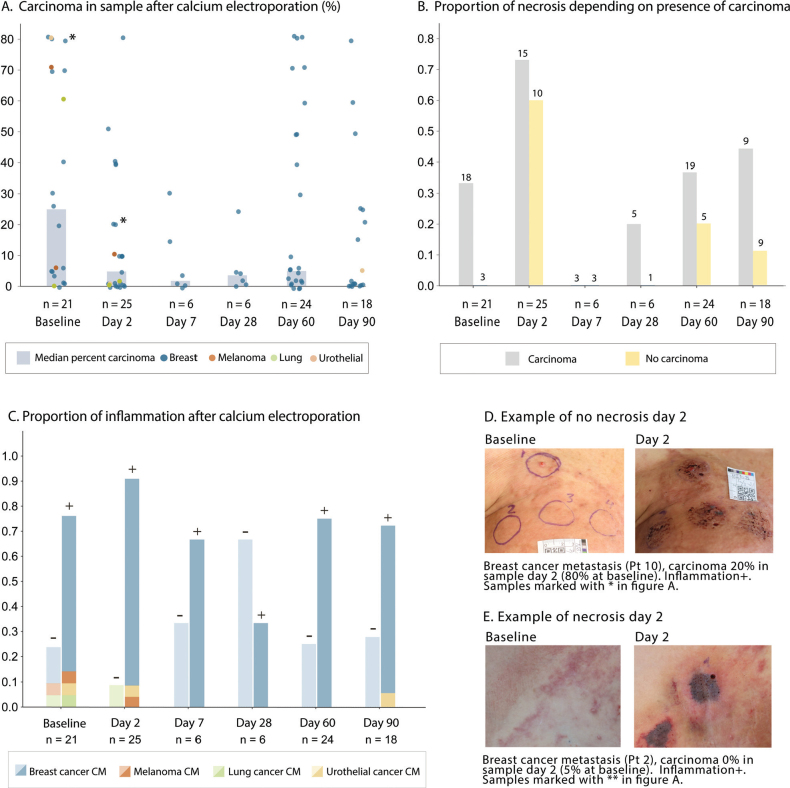
Evaluation of necrosis, carcinoma, and inflammation before and after calcium electroporation. Panel A. Percent carcinoma in tumours (dot plot) and proportion of samples with necrosis (bars) at each time point. Different colours represent different tumour-types (0–1 samples per tumour were taken at each time point). Panel B. Proportion of biopsied tumours with necrosis according to whether there was observed viable tumour cells in that sample (carcinoma % above 0). Panel C Proportion of inflammation after calcium electroporation. Presence of inflammation indicated by ±. Panel D Clinical photography of targets at baseline and after 2 days in patient with no necrosis and no clinical response. Panel E. Clinical photography of targets at baseline and after 2 days in patient with necrosis and clinical necrosis in treated areas.

Two days after treatment, necrosis was observed in 17 of 25 samples (68%) evaluated by histopathology and corresponded to findings from clinical inspection (Figure 3B, 3D and Supplementary Figure S4). A numeric reduction in the proportion of viable tumour cells in biopsied samples were noted to occur after CaEP treatment, with a low (<10%) degree of carcinoma observed in 58 and 61% in samples from day 60 and 90 respectively (Figure 3A). Inflammation was prevalent in a high proportion of samples throughout the study and was prevalent in 92% of sampled tumours on day 2 (Figure 3C).

### PD-L1

A total of 101 samples were analysed to assess PD-L1 expression. Of these, 96 samples were quantified for PD-L1 positivity (Figure 1 and Supplementary Figure S5). The remaining five samples exhibited areas of positive PD-L1 expression without the presence of carcinoma and were excluded from the analysis. These findings are noteworthy as they suggest the presence of PD-L1-positive cells in regions beyond the carcinoma areas.

There was no statistically significant change in PD-L1 expression over time in analysed samples (Figure 1 and Supplementary Figure S5).

### HER2

A total of 102 samples were analysed to assess HER2-positivity expressed as percentage of positive tumour cells in samples in increments of 1, 5 or 10. The highest median score of 3 [range 0–3] equal to high expression was observed on day 7 (*n* = 6). The median score at each time point is displayed in Table 3. There was clinical indication of an immunological effect in treated tumour lesions in three of four HER2+ patients, further described below.

**Table 3:** Baseline histology and clinical characteristics of patients who presented signs of clinical response or no response (see Figure 6).

Histopathology findings and response in select patients																	
	Baseline histology in patients with clinical sign of immune response													Patient with no clinical response			
*Patient no.*	Pt 2		Pt 11				Pt 13			Pt 15				Pt 10			
*Diagnosis*	Breast cancer		Urothelial cancer				Breast cancer			Breast cancer				Breast cancer			
*Previousirradiation*	Yes		Yes				Yes			Yes				Yes			
*Anatomical location*	Thorax		Thorax				Thorax			Thorax				Thorax			
*Biopsy site*	Edge		Edge				Edge			Centre				Edge			
*Mitosis*	No		Yes				No			No				Yes			
*Necrosis*	No		Yes				No			No				No			
*Inflammation*	No		Yes				Yes			Yes				No			
*TILs (% of cells in viable tumour stroma)*	0		30				80			0				3			
*Nuclear swelling*	Yes		No				Yes			No				No			
*Elastotic degeneration*	0		Yes				Yes			0				Yes			
*Carcinoma (% of sample)*	5		80				5			0				80			
*PD-L1 (% pos immune cells)*	2		20				30			NA				1			
*ER (% pos tumour cells)*	1		0				10			10				0			
*HER2 (% pos tumour cells)*	3		0				3			0				3			
*CD3 (% pos cells)*	40		20				10			10				10			
*CD4 (% pos cells)*	**40**		**10**				**30**			**30**				5*			
*CD8 (% pos cells)*	**10**		**10**				**10**			**10**				5*			
*CD20 (% pos cells)*	5		1				1			1				1			
*FOXP3 (% pos cells)*	10		5				10			5				10			
Histopathology findings by time point																	
Day	0 *N* = 21^a^			2 *N* = 26^a^		7 *N* = 6^a^		28 *N* = 6^a^			60 *N* = 24^a^		90 *N* = 21^a^			240 *N* = 1^a^	
	*n*	%		*n*	%	*n*	%	*n*	%		*n*	%	*n*		%	*n*	%
*TILs (% of cells in viable tumour stroma)*	3	0–80		0	0–40	5	0–60	1	0–10		1	0–40	1		0–30	1	1–1
*PD-L1 (% pos immune cells)*	2	0–30		0	0–40	1	0–40	0	0–1		0	0–50	1		0–30	0	0–0
*ER (% pos tumour cells)*	1	0–100		0	0–100	0	0–5	0	0–10		1	0–100	0		0–100	0	0–0
*HER2 (% pos tumour cells)*	0	0–3		0	0–3	3	0–3	0	0–3		0	0–3	0		0–3	1	1–1
*CD3 (% pos cells)*	10	1–50		8	1–50	10	5–10	5	5–10		5	1–30	5		1–50	1	1–1
*CD4 (% pos cells)*	20	1–60		28	1–80	35	30–60	35	20–60		10	1–50	10		5–50	5	5–5
*CD8 (% pos cells)*	5	1–30		5	1–30	5	1–10	3	1–10		5	1–30	5		1–20	1	1–1
*CD20 (% pos cells)*	1	0–5		1	0–10	0	0–10	3	0–10		0	0–10	0		0–10	0	0–0
*FOXP3 (% pos cells)*	5	1–10		1	0–10	5	1–10	5	0–10		5	0–10	1		0–10	1	1–1

a Median (range);

* : lower baseline value than all patients with clinical sign of immune response.

Pos: positive.

### Response

**Figure 4:**
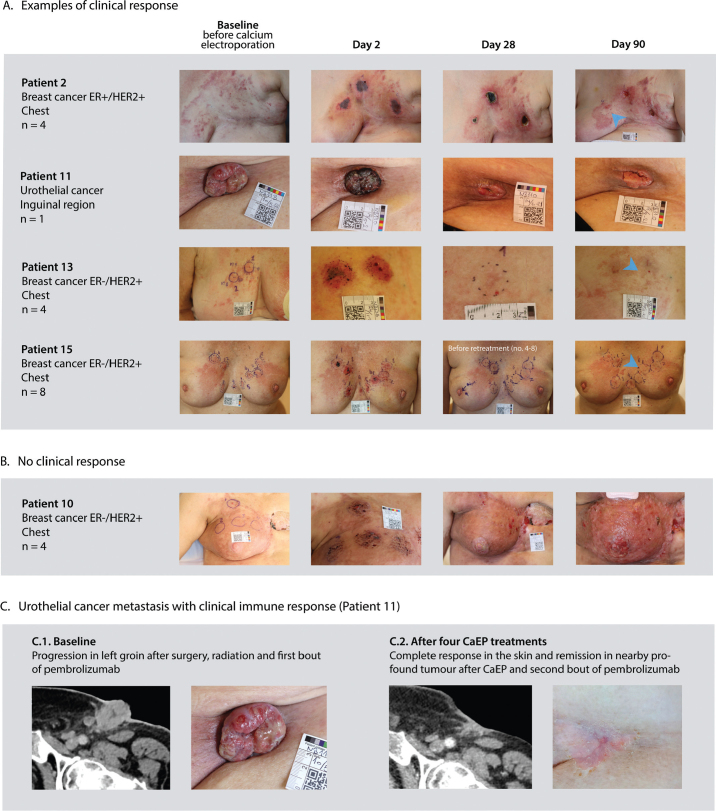
Examples of clinical response to calcium electroporation. (A) On day two, a characteristic demarcated necrosis was often observed with a narrow border of inflammation. On day seven, an eschar usually started to form, often still present on day 28. During the healing process, the eschar fell off, with a resulting scar or wound usually observed day 60. On day 90, a scar or healed skin could be observed in successfully treated areas. The blue arrow indicates areas where skin treated with calcium electroporation has minimal tumour infiltration at 3 months follow up. (B) Patient 10 exhibited no sign of response to treatment and had continued rapid progression of extensive breast cancer recurrence. Examples of tumour evolution across all treated patients are depicted in Supplementary Figure S4. (C) Urothelial cancer metastasis with clinical immune response to calcium electroporation. Images in C.1. and C.2. from a related case report, with permission, Open Access (Vissing et al., Acta Oncologica 2023) [28]

A total of 49 targets were treated. All included targets were cutaneous, and no subcutaneous targets were included. Clinical response (e.g., necrosis or tumour reduction) was observed in 16 of 17 patients, except for patient 10 (advanced ER-/HER2+ breast cancer recurrence, see Figure 4B). Response to CaEP was evaluated at one (*n* = 14), two (*n* = 29) and 3 months (*n* = 19) after treatment. Response evaluation at 1 month was not feasible in 8 of 24 tumours due to ulcerated- or crusted wounds. After 2 months, a response (≥30% decrease in diameter) was observed in 17 of 29 tumour lesions (58.6%) of which nine tumour lesions (31%) had a complete response. After 3 months, 14 of 19 tumour lesions (68.4%) showed a treatment response, of which 10 (53%) had a complete clinical response. This corresponds to an overall response rate of 28.5% from baseline (14 of 49 treated areas). Smaller tumour diameters were associated with a better treatment response at 3 months (–2.6%-point per mm, *p* = 0.006). In exploratory analysis of baseline markers associated with treatment response at 2 months, none of the predefined candidate variables showed significant association with the percentage change in tumour size in univariate analysis. None of the candidate variables were associated with the treatment response in logistic regression modelling.

### Clinical signs of immunological response

During follow-up, an interesting clinical observation was found in treated targets of patients 2, 11, 13, and 15, which displayed decrease in susceptibility to invasion from surrounding metastases, where skin remained disease free or had less progression than surrounding areas. This observation could suggest a potential localised protective effect. Interestingly, the patient with progressive urothelial cancer (Pt 11) showed complete response in the skin and remission of deeper untreated tissue at 12 months follow up after four CaEP treatments, without change in concomitant immunotherapy treatment regimen (pembrolizumab), as detailed in [28].

Table 3 summarises baseline histology and clinical characteristics of the patients where clinical signs of response and immune modulation was observed, as well as the one patient (Pt 10) where CaEP seemed not to have any effect. It also presents median values of immune markers at different time points for reference. Denoted on the table (*), patient 10 had a lower percentage of CD4 and CD8 positive cells from baseline tumour samples compared to the selected responding patients (Pt 2, 11, 13, 15), potentially indicating a difference in the immune profile or immune activity in that case. The patients with clinical signs of immune response following CaEP had higher CD8 expression (all 10%) than the median of 5% (1–30) and had all been previously irradiated in treated areas. The cases with clinical signs of immune response and the one case with no response to treatment are depicted in Figure 4, with histological properties shown in Table 3.

### Adverse events

Adverse events were recorded throughout the study. Four patients (no. 9, 12, 14, and 3) required hospitalisation for reasons not related to the study. One serious adverse event possibly related to CaEP was reported on day two, involving patient 16 who was admitted with bleeding from a biopsy site, due to chemotherapy-related thrombopenia. The patient was discharged within a few days and continued in the study.

After 2 months, one patient reported ulceration grade III associated with CaEP. Five cases of mild to moderate symptoms related to calcium were reported at 2 months, including mild suppuration (*n* = 1), moderate ulceration (*n* = 1), mild pain (*n* = 1), and pruritus (*n* = 2). For a full report of adverse events, Supplementary Table S3.

## Discussion

This exploratory study represents the first clinical investigation of the effect of CaEP on the tumour microenvironment in cutaneous metastases. Histological analysis was performed on of biopsy samples taken systematically before and after treatment. The primary outcome (change in TIL counts) was not significant; however, we observed that CaEP-induced inflammatory and necrotic tumour responses, supporting its potential effectiveness for cutaneous metastases.

CaEP is a novel treatment strategy with broad applicability in many cancers. Understanding its molecular and cellular effects is crucial, particularly for cutaneous metastases from different cancer types, as the tumour microenvironment may play a significant role in tumour control or cancer progression [29, 30]. We analysed lymphocyte subpopulations to better understand the interplay between the microenvironment and tumour evolution following local CaEP treatment.

We found potential predictors of immune modulation and changes in the microenvironment following treatment, including a decline in FOXP3 expression and a trend towards an increase in CD4-positive cells. These changes could indicate a shift towards a less immunosuppressive tumour microenvironment and a more active anti-tumour immune response [31–33]. This may have implications for enhancing the efficacy of immunotherapy in combination with CaEP [17, 34–36]. In the context of this study, the one patient in concomitant pembrolizumab treatment had improved response to systemic treatment after CaEP [28].

The treatment was well-tolerated with minimal adverse events, and most patients exhibited response with clinical tumour necrosis observed in 16 of 17 cases. We also noticed a trend towards reduced carcinoma levels after initial treatment and an anti-tumour effect. Necrosis was particularly prominent in biopsies taken 2 days after treatment, consistent with previous preclinical findings [1]. In a previous clinical study [13], necrosis was not observed in biopsies 7-days after treatment, which along with findings in this study suggest necrosis to be an acute-subacute response to CaEP, particularly visible shortly after treatment.

During follow-up, we observed reduced susceptibility to invasion from surrounding metastases in certain treated areas, possibly indicating a local immune effect similar to reports on irradiated cutaneous metastases [37]. This suggests that CaEP may induce immunogenic cell death, leading to immune system activation within the treated site. Notably, a preclinical study has established a connection between immune response and tumour necrosis-inducing treatment, highlighting its potential as an innovative strategy for cancer treatment [8].

Although this study was not designed for response evaluation, complete response was observed in 10 of 19 evaluable tumour lesions after 3 months, with higher response in smaller tumours. The overall response rate of 28% in an intention-to-treat analysis is comparable to a recent study involving CaEP for different types of cutaneous metastases measuring <3 cm in diameter [38].

In summary, our study highlighted encouraging clinical signs of immune response, which were unrelated to changes in TILs, in line with contemporary preclinical findings [10]. Notably, three patients with HER2 positive breast cancer metastases exhibited a possible protective effect in treated areas, echoing observations from a recent case report by Jensen et al. [16]. In addition, the signs of an abscopal response when CaEP was administered alongside immunotherapy in one of the participants suggests a potential for an immunostimulatory effect (recently published [28]). These compelling outcomes provide rationale for future investigations exploring the synergistic potential of CaEP in combination with immunotherapy for cancer management.

## Limitations

This study was a smaller, explorative study. The inclusion of mostly older patients with advanced stage IV disease might have introduced biases and limited the generalisability of the findings, as factors like immune senescence and systemic immunosuppression from tumour burden and prior treatments could have influenced the results [34, 36]. Additionally, baseline samples were derived from larger tumours, potentially leading to differences in histological properties compared to smaller tumours, which could have impacted the interpretation of the findings. The primary endpoint was not confirmed as evaluating TILs related to viable tumour tissue was compromised by extensive tumour necrosis and low carcinoma following CaEP treatment, which was also the case for evaluation of PD-L1-expression. As such, future studies could aim to study other immune cells of the tumour microenvironment following CaEP. Moreover, tumour heterogeneity obscured systematic biopsy procedures.

## Conclusion

In this exploratory study, the impact of CaEP treatment on tumour-associated immune cells in patients with skin metastases of different cancer types was investigated. The study supports CaEP as an anti-tumour treatment with evident tumour necrosis on day two. No change in TIL count, a decrease in FOXP3-positive cells and a trend towards an increase in CD4-positive cells were observed. Evaluating TILs and PD-L1 was complicated by the extensive tumour necrosis following CaEP. We observed clinical signs of response in 16 of 17 patients. Four patients showed signs of immune effect during follow-up, of which three had HER2 positive breast cancer metastases and the fourth had urothelial cancer metastasis. Future studies should aim to investigate CaEP in combination with immunotherapy.

## Supplementary Material




