# Supplementary material for: Immune cell populations in the tumour environment following calcium electroporation for cutaneous metastasis: a histopathological study
Source: Acta Oncol. 2024 May 28;63:19462. doi: 10.2340/1651-226X.2024.19462 (PMC11332521; doi:10.2340/1651-226X.2024.19462)
Supplement: Supplementary file 1 [file AO-63-19462-s1.pdf]

Supplementary material has been published as submitted. It has not been copyedited, or typeset by Acta Oncologica

Supplementary Figure 1.

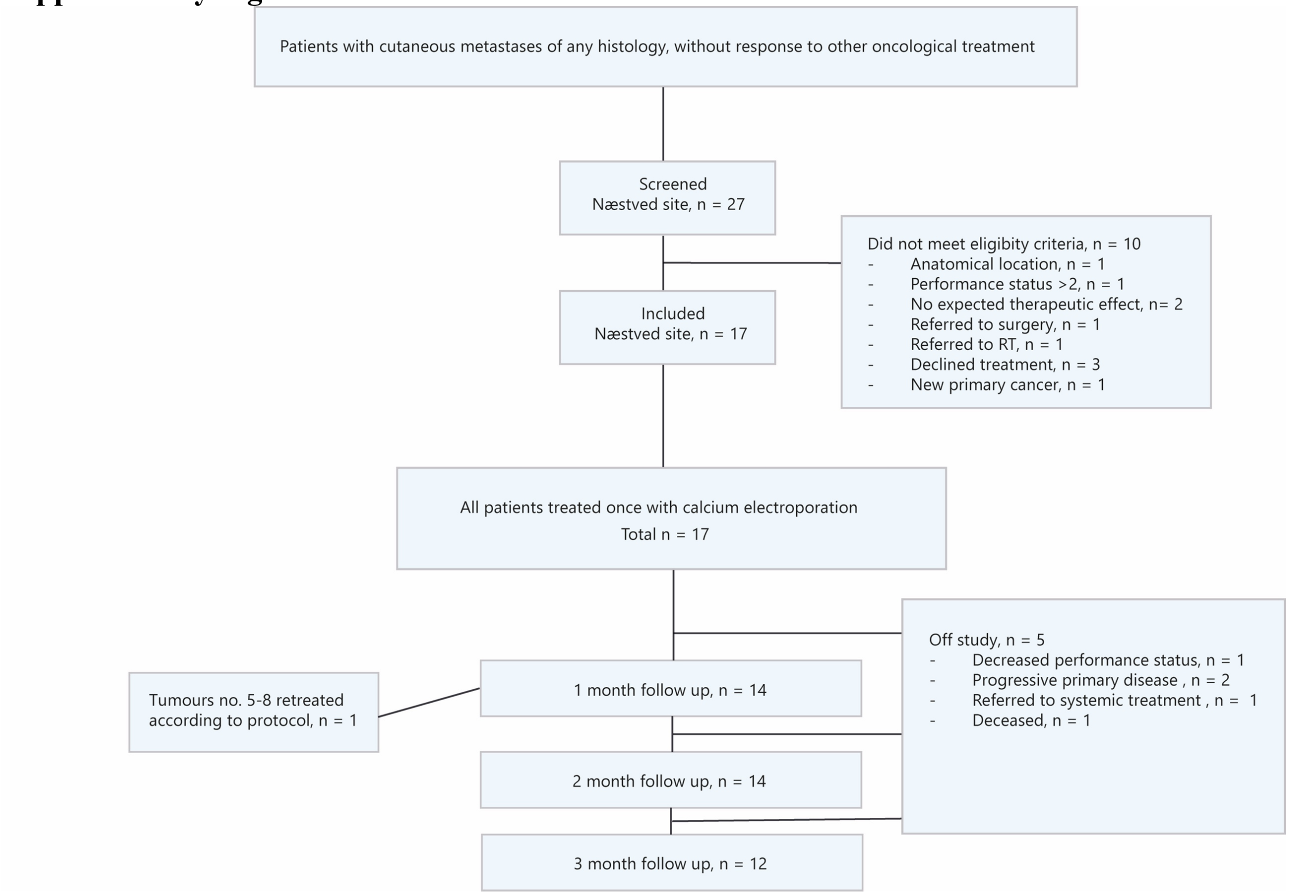

Supplementary Figure 2.

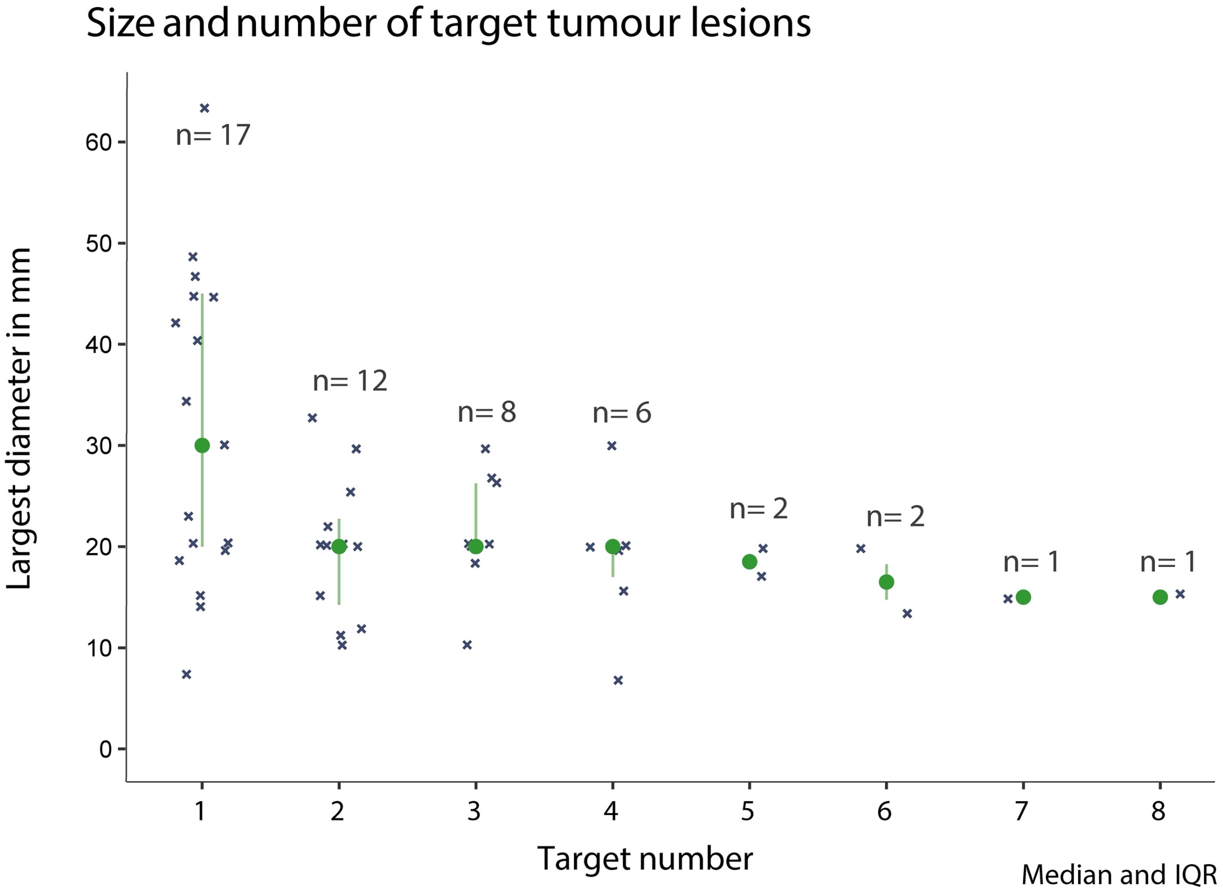

Supplementary Figure 3.

Examples of histological staining[1]

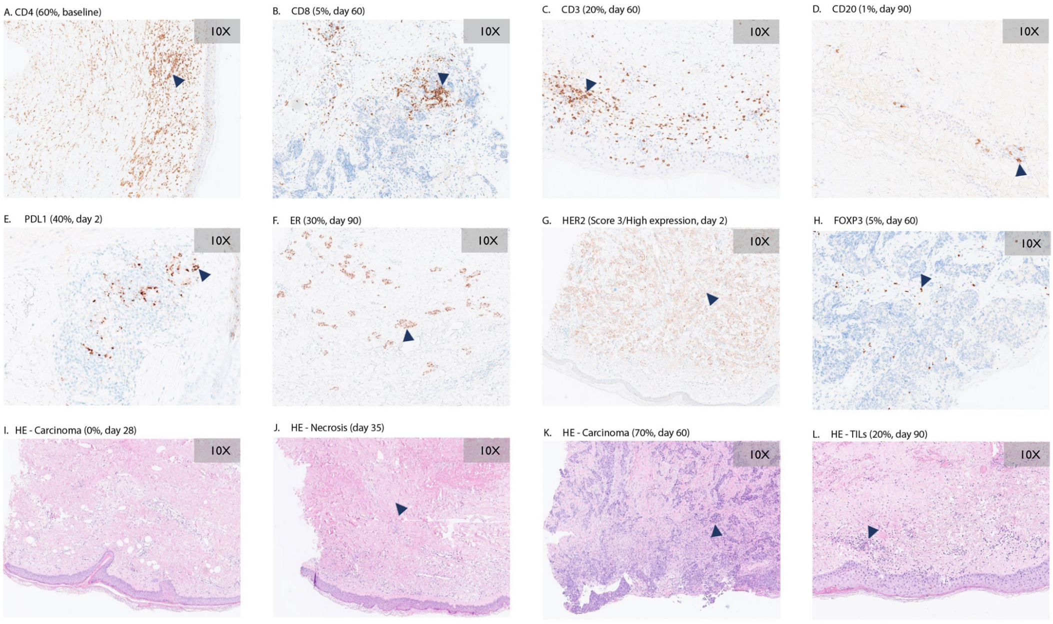

[1] Marker (measure, days from calcium electroporation)

Sample specifics: A. CD4 (60%, day 0, Pt 16, sample no. 101). B. CD8 (5 %(hotspot), Pt 15, day 60, sample no. 102). C. CD3 (20%, Pt 15, day 60, sample no. 108). D. CD20 (1%, Pt 15 , day 90 , sample no. 107). E. PDL1 (40%, Pt 13, day 2, sample no. 73). F. ER (30%, Pt 15, day 90, sample no. 107) G. HER2 (Score 3/High expression, Pt 15, day 2, sample no. 93). H. FOXP3 (5%, Pt 15, day 60, sampleno. 102). I. HE - Carcinoma (0%, Pt 15, day 28, sample no. 96) J. HE - Necrosis (Pt 15, day 35, sample no. 100). K. HE - Carcinoma (70%, Pt 15, day 60, sample no. 102). L. HE - TILs (20%, Pt 15, day 90, sample no. 108)

Supplementary Figure 4.

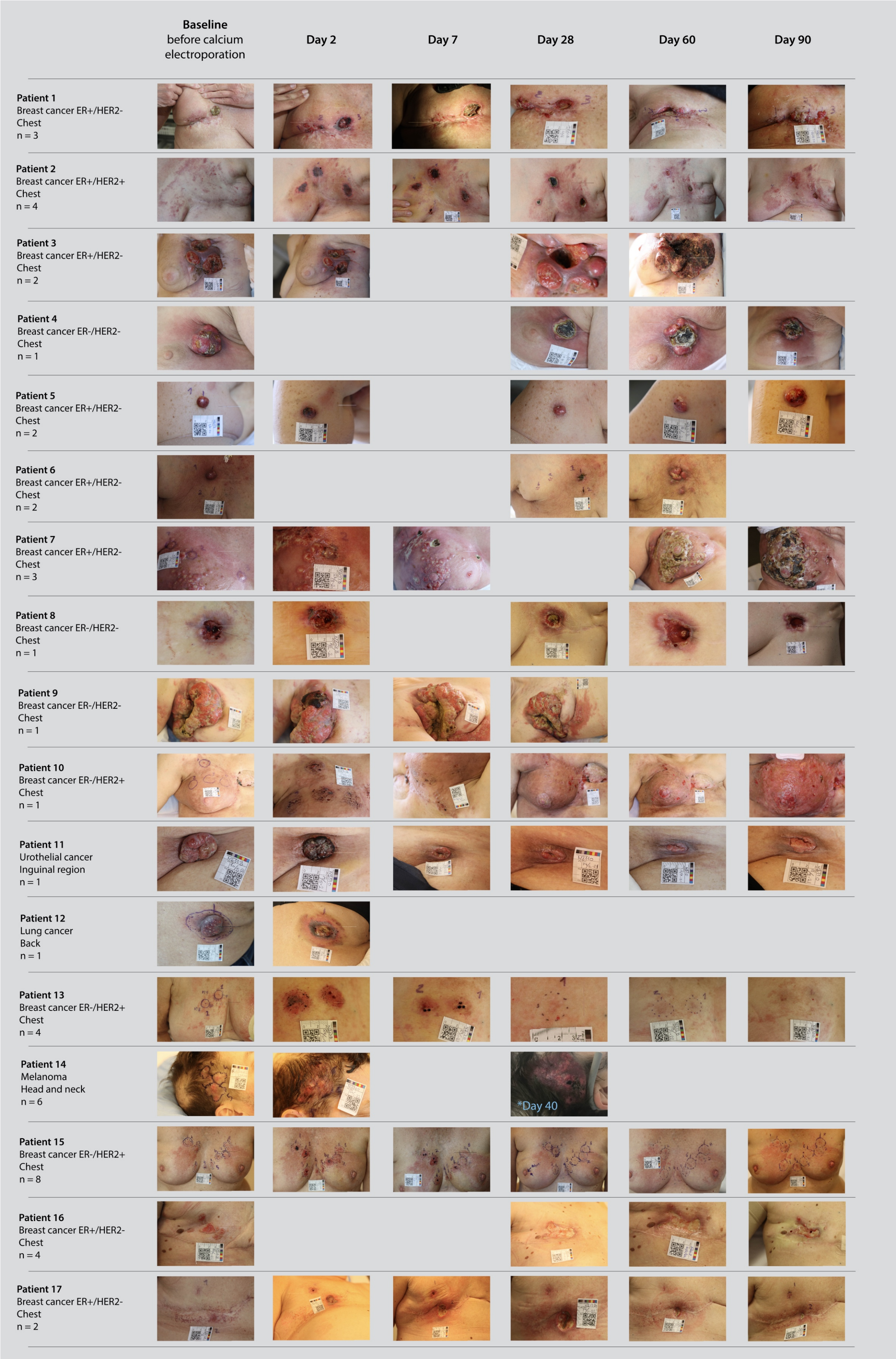

Supplementary Figure 5.

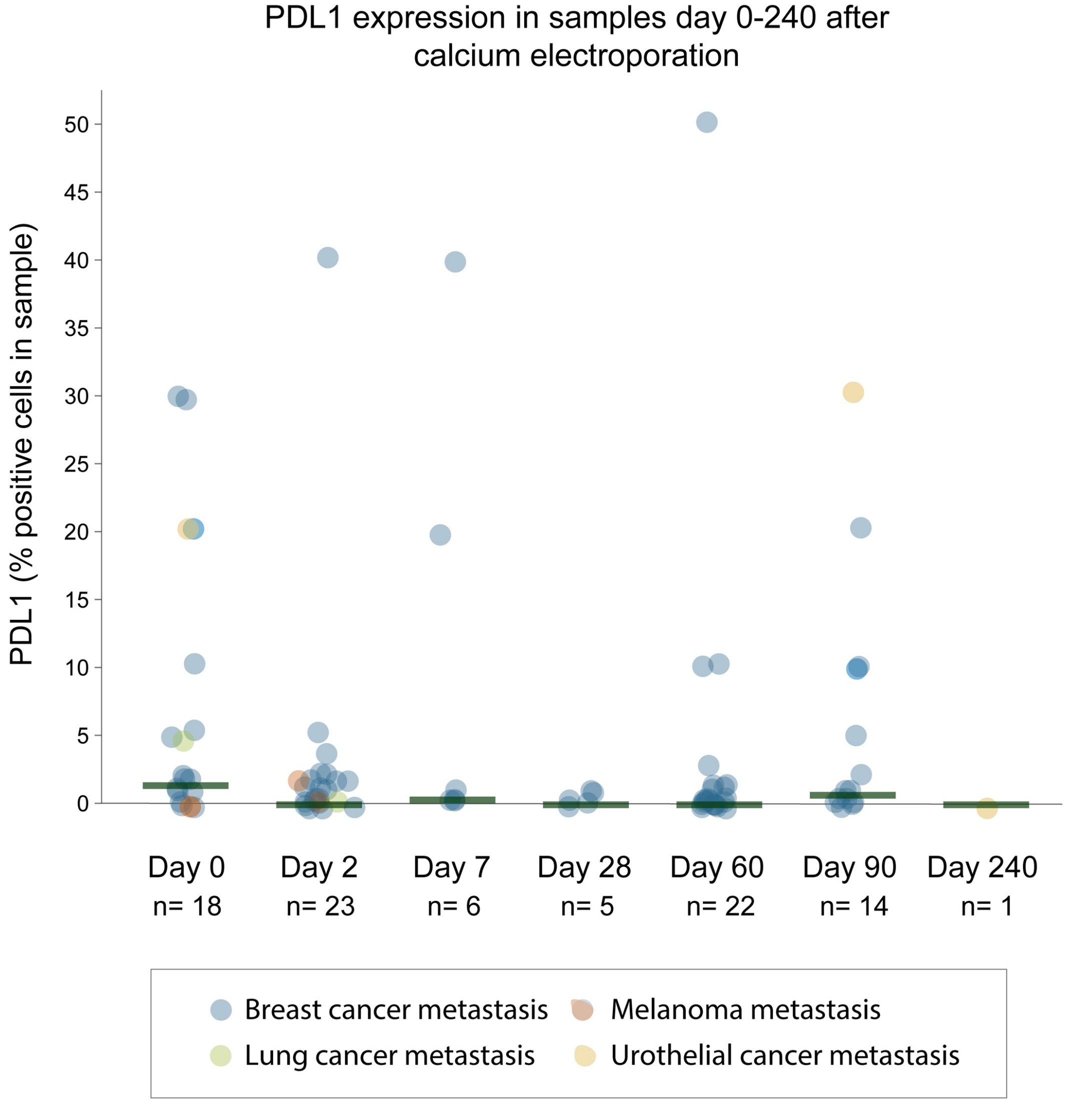

Supplementary Figure 6.

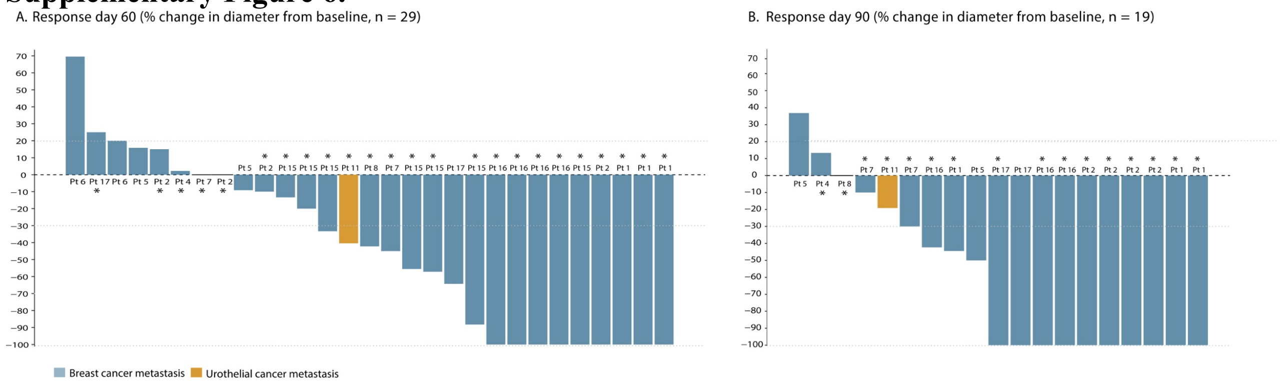

Supplementary Figure 7.

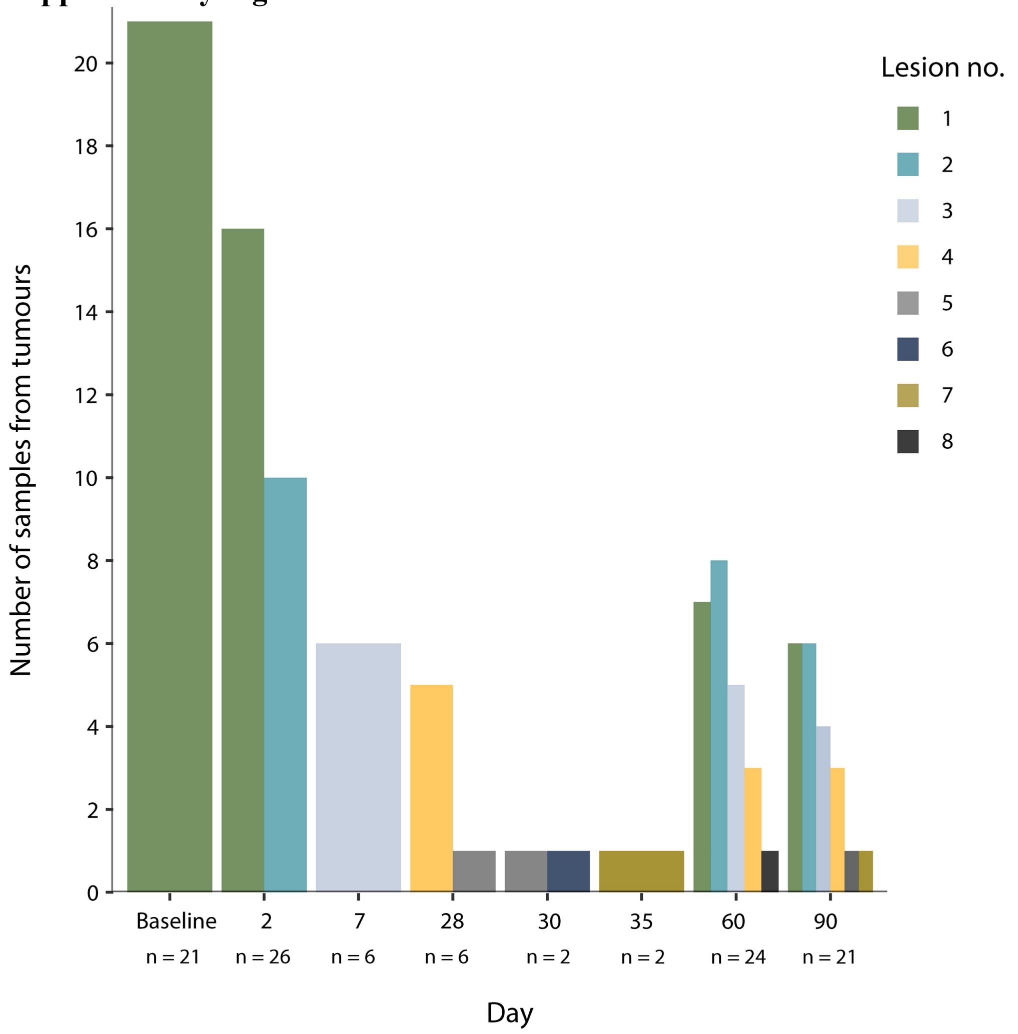

Supplementary Figure 8.

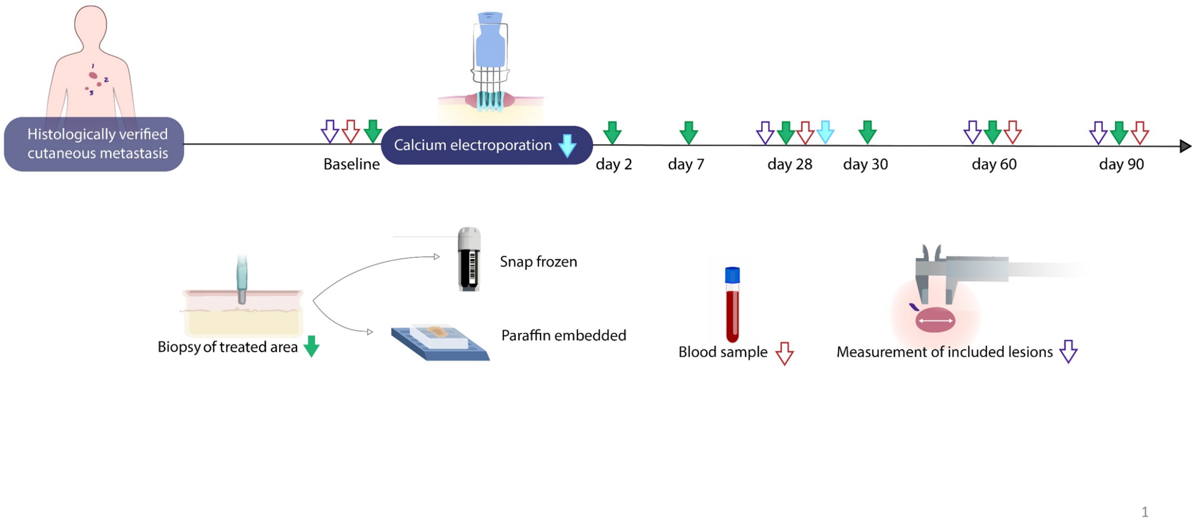

Supplementary Figure 9.

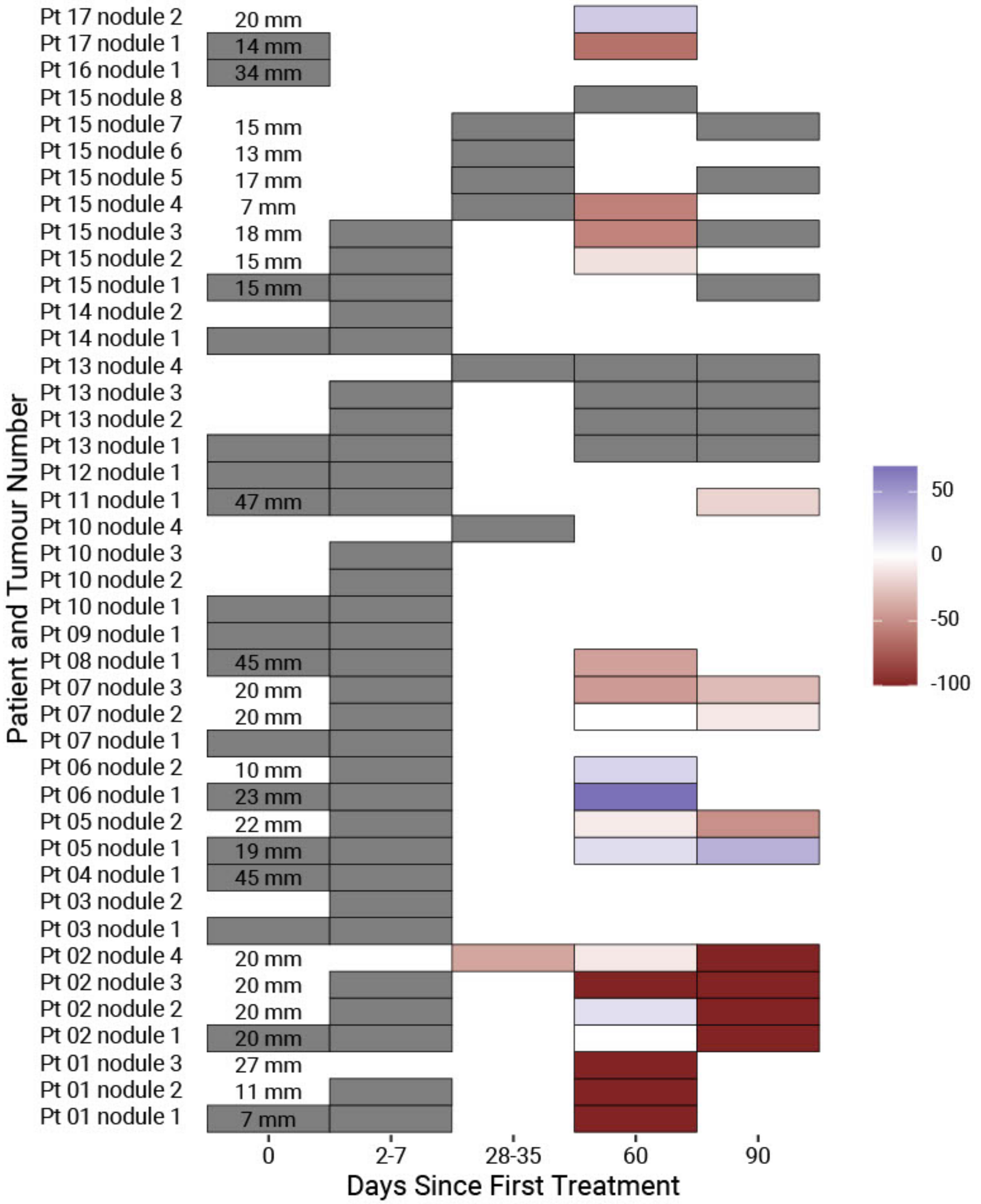

Grey boxes denote a tumour biopsy where data on change in size relative to baseline was not recorded.
Other fill colors of boxes denotes percent change in tumour size from baseline.
The blank area represent potential test days on which the nodule was not biopsied.
The text on the plot above day 0 gives the size of that specific tumour at baseline
