# Supplementary material for: Immune cell populations in the tumour environment following calcium electroporation for cutaneous metastasis: a histopathological study
Source: Acta Oncol. 2024 May 28;63:19462. doi: 10.2340/1651-226X.2024.19462 (PMC11332521; doi:10.2340/1651-226X.2024.19462)
Supplement: Supplementary file 2 [file AO-63-19462-s2.pdf]

Supplementary material has been published as submitted. It has not been copyedited, or typeset by Acta Oncologica

Table S1. CaEP-B Biopsy plan. If a patient only had three treatable tumours (or only requested treatment of three tumours), the chart was followed from no. 1-3. CaEP: Calcium electroporation; B: Biopsy, paraffin embedded. (F)/ (B): Biopsy if possible (Day 0: Biopsy if tumour > 2 cm largest diameter); F: Frozen biopsy 2 mm. (F): Biopsy if possible (Day 0: Biopsy if tumour > 2 cm largest diameter). *Note tumour no. in a single patient.

Tumour no.*	Day 0	Day 2	Day 7	Day 28	Day 30	Day 35	Day 60	Day 90
**1**	(F) + B + CaEP	B					(B)	(B)
**2**	CaEP	B + F			(F)		(B)	(B)
**3**	(B) + F + CaEP		B				(B)+(F)	(B)
**4**	CaEP		F	B	(F)		(B)	(B)
**5**	CaEP			B + CaEP	B		(B)+(F)	(B)
**6**	CaEP			CaEP	B+F		(B)	(B)+(F)
**7**	CaEP			F + CaEP		B	(B)	(B)+(F)
**8**	CaEP			CaEP			B+F	B+F

Table S2. HER2 and ER expression in samples day 0-90. HER2 expression score (0-3): Normal (0-1), borderline (2), and high (3). NA: Not applicable. ER (oestrogen receptor); HER2 (human epidermal growth factor receptor 2).

	Baseline	Day 2	Day 7	Day 28	Day 60	Day 90
**Samples from breast cancer metastases**	n = 16	n = 21	n = 6	n = 6	n = 24	n = 20
HER2 expression						
Normal	10 (62%)	16 (80%)	2 (34%)	4 (67%)	20 (84%)	13 (77%)
Borderline	3 (19%)	0 (0%)	0 (0%)	0 (0%)	3 (12%)	3 (18%)
High	3 (19%)	4 (20%)	4 (67%)	2 (33%)	1 (4.2%)	1 (5.9%)
NA (n)	0	1	0	0	0	3
ER expression						
Mean % (range)	10 (0, 40)	0 (0, 0)	0 (0, 4)	0 (0, 4)	1 (0, 35)	0 (0, 30)
NA (n)	1	1	0	0	1	4
**Samples from other cancer metastases**	n = 5	n = 5	-	-	-	n = 1
HER2 expression						
Normal	5 (100%)	5 (100%)	-	-	-	1 (100%)
Borderline	0 (0%)	0 (0%)	-	-	-	0 (0%)
High	0 (0%)	0 (0%)	-	-	-	0 (0%)
NA (n)	0	0	-	-	-	0
ER expression						
Mean % (range)	0 (0, 0)	0 (0, 0)	-	-	-	30 (30, 30)
NA (n)	0	0				0

Table S3. Skin tumour related CTCAE v. 4.0 scores at baseline and two months after calcium electroporation.

ADVERSE EVENTS AFTER CALCIUM ELECTROPORATION					
**ODOR**		Baseline before treatment, n=17		Two months, n = 12	
**1**	None	10	59%	7	58%
**2**	Mild odour: physician intervention not indicated; self-care interventions	4	24%	4	33%
**3**	Pronounced odour; psychosocial impact; patient seeks medical intervention	3	18%	1	8%
**SKIN INFECTION**					
**0**	None	16	94%	12	100%
**1**	Localized; local intervention indicated (e.g., topical antibiotic, antifungal, or antiviral)	0	0%	0	0%
**2**	Localized; oral intervention indicated (e.g., oral antibiotic, antifungal, or antiviral)	1	6%	0	0%
**3**	Localized; local intervention indicated (e.g., topical antibiotic, antifungal, or antiviral)	0	0%	0	0%
**4**	Life-threatening consequences: urgent intervention indicated	0	0%	0	0%
**PAIN**					
**0**	None	8	47%	6	50%
**1**	Mild	7	41%	4	33%
**2**	Moderate pain; limiting instrumental ADL	3	18%	1	8%
**3**	Severe pain; limiting self-care ADL	0	0%	0	0%
**HYPERPIGMENTATION**					
**0**	None	17	100%	11	92%
**1**	Hyperpigmentation covering <10% BSA; no psychosocial impact	0	0%	1	8%
**2**	Hyperpigmentation covering >10% BSA; associated psychosocial impact	0	0%	0	0%
**ULCERATION**					
**0**	None	5	29%	5	42%
**1**	1=Combined area of ulcers <1 cm; non-blanchable erythema of intact skin with associated warmth or oedema	1	6%	1	8%
**2**	Combined area of ulcers 1 - 2 cm; partial thickness skin loss involving skin or subcutaneous fat	3	18%	3	25%
**3**	Combined area of ulcers >2 cm; full-thickness skin loss involving damage to or necrosis of subcutaneous tissue that may extend down to fascia.	7	41%	7	58%
**4**	Any size ulcer with extensive destruction, tissue necrosis, or damage to muscle, bone, or supporting structures with or without full thickness skin loss.	1	6%	1	8%
**SUPPURATION**					
**0**	None	7	41%	2	17%
**1**	Mild symptoms: intervention not indicated	6	35%	6	50%
**2**	Moderate; minimal intervention indicated, limiting age-appropriate ADL	4	24%	4	33%
**3**	Severe; hospitalization indicated disabling, limiting self-care ADL	0	0%	0	0%
**4**	Life-threatening, disabling	0	0%	0	0%
